# miR-122 inhibition in a human liver organoid model leads to liver inflammation, necrosis, steatofibrosis and dysregulated insulin signaling

**DOI:** 10.1371/journal.pone.0200847

**Published:** 2018-07-19

**Authors:** Hossein Sendi, Ivy Mead, Meimei Wan, Marjan Mehrab-Mohseni, Kenneth Koch, Anthony Atala, Herbert L. Bonkovsky, Colin E. Bishop

**Affiliations:** 1 The Laboratory for Liver Diseases and Metabolic Disorders, Section on Gastroenterology, Department of Internal Medicine, Wake Forest Baptist Medical Center, Winston-Salem, NC, United States of America; 2 Wake Forest Institute of Regenerative Medicine, Winston-Salem, NC, United States of America; University of Navarra School of Medicine and Center for Applied Medical Research (CIMA), SPAIN

## Abstract

To investigate the role of miR-122 in the development and regression of non-alcoholic fatty liver disease (NAFLD) *in vitro*, we used multicellular 3D human liver organoids developed in our laboratory. These organoids consist of primary human hepatocytes, Kupffer cells, quiescent stellate cells and liver sinusoidal endothelial cells. They remain viable and functional for 4 weeks expressing typical markers of liver function such as synthesis of albumin, urea, and alpha-1 p450 drug metabolism. Before mixing, hepatic cells were transduced with lentivirus to inhibit miR122 expression (ABM, CA). Immediately after the organoids were fully formed (day 4) or after 1 or 2 weeks of additional incubation (days 11 or 18), the organoids were analyzed using fluorescent live/dead staining and ATP production; total RNA was extracted for qPCR gene expression profiling. Our results show that miR-122 inhibition in liver organoids leads to inflammation, necrosis, steatosis and fibrosis. This was associated with increase in inflammatory cytokines (IL6, TNF), chemokines (CCL2, CCL3) and increase in a subset of Matrix Metaloproteinases (MMP8, MMP9). An altered expression of key genes in lipid metabolism (i.e LPL, LDLR) and insulin signaling (i.e GLUT4, IRS1) was also identified. *Conclusion*: Our results highlight the role of miR-122 inhibition in liver inflammation, steatofibrosis and dysregulation of insulin signaling. Patients with NAFLD are known to have altered levels of miR-122, therefore we suggest that miR-122 mimics could play a useful role in reversing liver steatofibrosis and insulin resistance seen in patients with NAFLD.

## Introduction

Primary human hepatocytes (PHH) when maintained in conventional 2D monolayer cultures de-differentiate and rapidly lose hepatocyte-specific functions [1]. In addition, such cultures only partially mimic the microenvironment of the liver because they lack other major hepatic cells and cellular microarchitecture. In recent years, liver organoids, three-dimensional aggregates of cells derived from the liver, have been developed and have been found to have several desirable properties (e.g., long cell lives with differentiated cell phenotypes, physiologic responses to perturbations) and to better reflect the normal physiology of the human liver[2]. Liver organoids, which are cellular 3D aggregates generated by stirring in bioreactors or gravitational aggregation in hanging drop cultures or on ultralow attachment (ULA) surfaces, constitute models that allow high-throughput screenings in 96-well format and studying different liver diseases in 3D liver models *in vitro*[3].

Chronic liver diseases of diverse etiologies are among leading causes of mortality and morbidity around the world. Chronic liver disease progresses through different pathological stages, which can eventually lead to advanced hepatic fibrosis and cirrhosis. Liver fibrosis is characterized by excessive extracellular matrix (ECM) deposition and fibrous scar formation, which is mainly due to the activation and transition of hepatic stellate cells [HSC, also known as Ito or fat-storing cells] to myofibroblasts in response to fibrogenic stimuli. It is also known that liver inflammation plays a major role in development of hepatic fibrosis. During the progression of fibrosis, injury-induced inflammatory responses trigger the recruitment of macrophages into the liver, where they produce cytokines and chemokines to induce the transition of HSCs into myofibroblasts that produce ECM [4]. For example, the cytokine CCL2, secreted by Kupffer cells and HSCs, plays an important role in this process. Nonalcoholic steatohepatitis (NASH), which is characterized by the presence of hepatic steatosis and inflammation with hepatocyte injury (ballooning) with or without fibrosis is a more advanced stage of fatty liver diseases that has reached epidemic proportions world-wide-with a global prevalence estimated as high as 25% [5,6]. Inflammation plays a major role in hepatic injury in NASH. Consistent with this notion, it was recently found that pharmacological inhibition of CCL2 can decrease hepatic inflammation and steatohepatitis in animal models of NASH [7].

Several micro RNAs (miRNAs) have recently been implicated in the development of liver fibrosis, with many of them affecting different steps of fibrogenesis [fibrosis]. One such miRNA, namely, miR-122, which is the most abundant miRNA within liver [8] has been suggested as potential systemic therapy for treatment of hepatic fibrosis [9]. It has been shown that levels of miR-122 are down-regulated in CCl4-induced liver fibrosis models in mice. Systemic injection of miR-122-expressing lentivirus increased miR-122 levels and reduced the amount of ECM components in the livers of CCl4-treated mice [10]. miR-122 has been suggested to play a key role in lipid accumulation in the liver by reducing YY1 mRNA stability to upregulate FXR-SHP signaling [11]; its decreased expression upregulates modulators of tissue remodeling (HIF-1α, vimentin and MAP3K3) in experimental NASH and fibrosis [12]; targets SIRT6 impacting on metabolic genes and fatty acid β-oxidation [13]. Also, it was shown that mice with deficiency in miR-122 can develop steatohepatitis, fibrosis and hepatocellular carcinoma. It is also known that miR-122 expression is reduced in patients with NASH, and in a subset of hepatocellular carcinomas [14].

Based on the known role of miR-122 deficiency in inducing hepatic steatofibrosis in animal models and based on the fact that patients with NASH are known to have altered levels of miR-122 in serum and liver, we decided to investigate the role of miR-122 in the development and regression of non-alcoholic fatty liver disease (NAFLD) in vitro. To this end, we generated a 3D human liver organoid model in our laboratory to study the effects of miR-122 inhibition in liver organoids.

## Materials and methods

### Construction of liver organoids

Cryopreserved, primary human hepatocytes, Kupffer cells and hepatic stellate cells were purchased from TRL/Lonza (NC USA). Liver sinusoidal endothelial cells were purchased from ScienCell (CA USA) and expanded for 3 passages according to the manufacturer’s instructions, before being cryopreserved in 5x105 cell aliquots. After thawing, cells were mixed in the following percentages: hepatocytes 80%, Kupffer 10%, stellate 5% and endothelial cells 5% using Clonetics complete hepatocyte growth medium (HCM), without hydrocortisone or amphotericin B, (Lonza, USA) containing 20% fetal bovine serum (Hyclone USA). Organoids, containing 1,200 total cells, were formed using gravitational aggregation in either InSphero hanging drop plates (1,200 cells/40ul) (InSphero, ME) or ultralow adhesion U bottomed plates (ULA) (1,200 cells/100ul) (Corning, USA). After 4 days for aggregation and compaction, organoids, formed using hanging drop technology, were transferred to 200uL serum-free medium in 96 well plates and were fed by removing 50% of the medium and replacing it with fresh serum-free medium every other day. Organoids formed in 96 well ULA plates were initially fed by adding 100uL serum-free medium HCM media, then fed every other day as above. Liver organoids formed by either method performed similarly.

### Histology and immunohistochemistry

Organoids were collected, washed twice in PBS, fixed for 20 min at room temperature in 4% paraformaldehyde and then embedded in HistoGel (ThermoFisher, USA) followed by paraffin. Sections of 5 μM were cut, deparaffinized and H&E and Sirius Red staining was performed according to standard protocols.

For immunohistochemistry, after rehydrating sections, endogenous peroxidase activity was removed by incubation in 3% H2O2 for 20 min. Antigen retrieval was performed in 10mM citrate buffer pH6 at 100o for 25 min, followed by blocking with DAKO protein block for 30min at room temperature. Slides were incubated with primary antibodies at 4o overnight, followed by incubation with horseradish peroxidase conjugated secondary antibodies for 1 hour. After washing, 0.05% DAB solution (3,3’-diaminobenzidine) was added for approximately 10 minutes, washed and counterstained with hematoxylin and visualized on a Zeiss Axiovert 200M microscope.

For whole mount immunohistochemistry, fixed organoids were permeabilized with 0.1% triton in PBS for 20 min then blocked with DAKO protein block for 2 hours, at room temperature. Organoids were incubated with primary antibodies overnight at 4o, washed 4 times in PBS and incubated at 4o overnight with secondary antibodies. Finally, organoids were washed 4 times in PBS, counterstained with DAPI (300nM in PBS) and imaged on a Leica TCS LSI macro confocal.

### Antibodies used

Primary antibodies against CD68, Connexin32, Cytokeratin, E-cadherin, human serum albumin, P450 reductase and Vimentin were purchased from Abcam (MA, USA). Donkey anti Rabbit:HRP and Goat anti mouse HRP were purchased from Jackson Immuno Research (PA, USA) while Goat anti Rabbit IgG: AF595 and Goat anti Mouse IgG were purchased from Life Technologies (CA, USA).

### Transmission electron microscopy

One day after formation organoids were fixed in 2.5% glutaraldehyde for 1 hour at room temperature and then processed and imaged for standard TEM by the WFUHS microscopy core.

### Viability and liver marker secretion

Viability was directly visualized using a LIVE/DEAD™ Viability/Cytotoxicity Kit (Invitrogen, USA). Oganoids were incubated for 5 minutes in a solution of green-fluorescent calcein AM and red-fluorescent ethidium homodimer 1, according to the manufacturer’s instructions, then visualized using a Leica macro confocal. In this assay, live cells stain green and dead cells stain red. For infected organoids, we used Calcein Blue and ethidium homodimer for staining of live and dead cells, respectively, since organoids infected with lentivirus expressed GFP as green color. Cell viability was also determined by measuring the ATP content of single organoids with the CellTiter-Glo Luminescent Cell Viability Assay (Promega, USA). Urea, human albumin, a1 antitrypsin and TNFα were measured using standard colormetric ELISA kits according to the manufacturer’s instructions. Urea and a1antitrypsin kits were purchased from AbCam, UK; Human specific serum albumin kits from Alpha diagnostics Int, USA and TNFa kits from thermos/Fisher, USA.

### Evaluation of drug metabolism

Standard multicellular organoids (1,200 cells/ organoid) were formed in round bottomed 96 well plates. For comparison, 2D cultures were formed by plating 105 cells (in the same proportions used to form the organoids) on matrigel coated flat bottomed 96 well plates to form a confluent layer. Drug toxicity in the organoids and monolayer cultures was then assessed by inducing cytochrome P450 activity using a mixture of rifampicin (25mM), 3-methylcholanthrene (3.78μg/mL), and phenobarbital (58.0μg/mL) (Sigma) in HCM medium (Lonza), inducing the cells for 24 hours. Then diazepam was added (2.5μg/mL) in HCM medium for 24 hours. Diazepam metabolites temazepam, nordiazepam, and oxazepam were measured in the cell supernatant using a Quantum Discovery Max triple quadrupole mass spectrometer (Thermo/Fisher)

### Toxicity assays

Troglitazone, in concentrations between 0 and 1mM, was added to 14 day liver organoids. After 48 hours, organoid viability was determined by cellular ATP content as above. Steatosis, the intracellular accumulation of neutral lipids, was measured qualitatively using the LipidTox Neutral Lipid stain kit (Thermo/Fisher, USA). Phospholipidosis, the intracellular accumulation of phospholipids, was measured qualitatively using the LipidTox Phospholipidosis detection stain kit (Thermo/Fisher, USA). Imaging was performed using the Leica macro confocal.

### Lentivirus infection

Appropriate proportions of hepatocytes, Kupffer cells, stellate cells, endothelial cells were mixed (80%, 10%, 5%, and 5%, respectively) and plated in 96 well plates. Every well of 96-well plates contained 1200 cells in total. Lentivirus (10^7^ pfu/ml) expressing Lenti-mir-Off ctrl vector or miR-122-Off lentivirus (ABM, Canada) was added to the cells immediately after mixing the cells with the respective Multiplicity of Infection (MOI = 2, 3 or 5). Either immediately after organoid made (day 4 after mixing or W0) or one week after organoid made (day 11 after mixing or W1) the organoids from each respective 96 well plate was collected and pooled for IHC or RT PCR.

### Real time PCR

Total RNA was extracted with miRNAeasy kit (Qiagen, USA). The cDNA was made using RT2 First Strand kit (Qiagen, USA) and mixed with RT2 SYBR Green/ROX qPCR Master Mix (Qiagen, USA) and the mixture was added into a 96-well RT2 fibrosis PCR Array (SABiosciences, USA) that contained primers for 84 key genes in fibrosis and 5 housekeeping genes according to manufacturer’s instruction. Thermal cycling was performed using ABI-7500 (Applied Biosystems, USA) with an initial denaturation at 95°C for 10 minutes, 40 cycles at 95°C for 15 seconds, and 60°C for 1 minute. Values of cycle threshold (Ct) obtained in quantification were used for calculations of fold changes in mRNA abundance using 2-ΔΔCt method. Data was analyzed using RT2 profiler PCR array data analysis version 3.5 (SABiosciences, USA).

## Results

### Characterization of liver organoids

**Fig 1:**
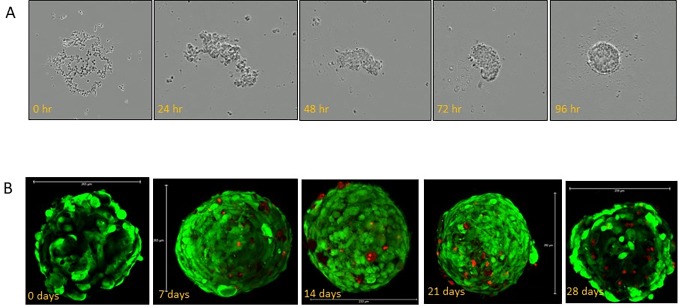
Construction of liver organoids. (A) shows aggregation and compaction of cells at 24 hr. intervals. Organoids were fully formed after 4 days undisturbed culture. (B) Direct viability visualization of organoids over a 28-day period using calcein AM to stain live cells green and ethidium homodimer to stain dead cells red. Even by 28 days, the percentage of dead cells was <10%.

**Fig 2:**
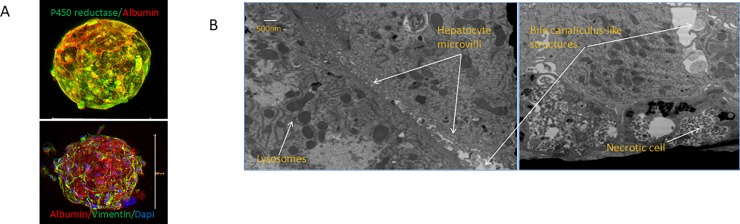
Liver Organoid characterization. (A) whole mount staining shows co-expression of p450 reductase (green) and serum albumin (red). Expression of hepatocyte marker serum albumin and stellate cell marker vimentin indicates that the latter cells are present tightly interspersed with hepatocytes. (B) Transmission electron microscopy of newly formed liver organoids (day 2 after formation) shows, even at this early stage, the presence of rudimentary bile canaliculi in between adjacent hepatocytes indicating the establishment of a primitive biliary system.

As shown in Fig 1A the multicellular liver organoids aggregated and formed a compact organoid of approximately 205uM after 4 days of culture. Live/dead staining shows excellent viability for at least 28 days (Fig 1B). Liver organoid were fully characterized as previously shown [15]. Whole mount staining shows co-expression of p450 reductase (green) and serum albumin (red, Fig 2A). Expression of hepatocyte marker serum albumin and stellate cell marker vimentin indicates that the latter cells are present tightly interspersed with hepatocytes. Transmission electron microscopy of newly formed liver organoids (day 2 after formation) shows, even at this early stage, the presence of rudimentary bile canaliculi in between adjacent hepatocytes indicating the establishment of a primitive biliary system (Fig 2B).

**Fig 3:**
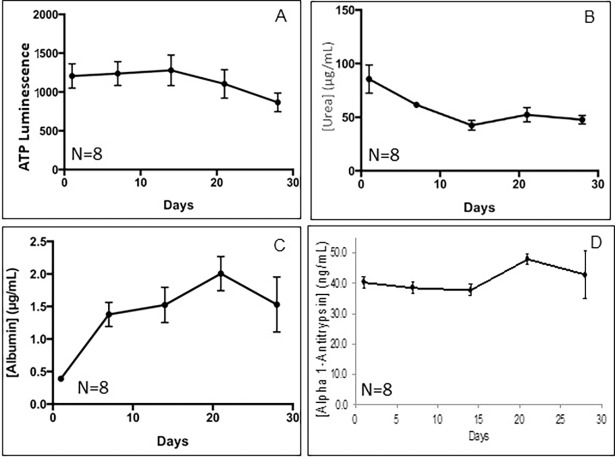
Expression of liver markers during long-term culture. (A) Cellular ATP expression indicates that viability only drops by approximately 10% over 28days. (B) Although albumin levels excreted into the medium are initially relatively low, expression triples by 7 days and thereafter remains high over the entire 28 day period. (C) Urea synthesis dropped somewhat but remained at over 50ug/ml at day 28. (D) Hepatocyte alpha-1 antitrypsin levels remain constant over the entire 28 day period.

As shown in Fig 3 the functionality of the liver organoids was sustained over long term culture (28 days). Viability, as measured by cellular ATP only dropped by approximately 10% over 28 days in culture. Urea, albumin and hepatocyte alpha-1 antitrypsin were easily detected and remained high over the entire culture period.

To evaluate drug metabolism, cytochrome P450 enzymes were induced using rifampicin, 3-methylcholanthrene, and phenobarbital. Subsequently, organoids were exposed to diazepam, which is converted into primary metabolites temazepam and nordiazepam primarily by CYP3A4 and CYP2C19, and a secondary metabolite, oxazepam (S1 Fig).2 Liver organoids were found to have measurable cytochrome P450 drug metabolism activity for at least 28 days, in comparison to standard monolayer sandwich cultures that lost CYP450 activity after 7 days (S1 Fig). It is also important to note again the difference in total cell number between the 3D culture model (~1.2 x 10^3^ cells/sample) and the 2D culture model (10^5^ cells/sample).

As a further indication of functionality, we tested the ability of the organoids to detect the toxicity of the drug Troglitazone. This drug was initially approved as an anti-diabetic but soon withdrawn from the market due to liver toxicity. As shown in S2A Fig, an EC50 value of approximately 100uM was obtained. S2B Fig shows the intracellular accumulation of neutral lipids after troglitazone insult as a model for liver steatosis and S2C Fig intracellular accumulation of phospholipids modelling liver phospholipidosis.

### Lentivirus expressing miR-122 inhibitors are most effective on liver organoids when added to the media immediately after co-culturing different liver cells

To study the effects of miR-122 inhibition on the liver organoids, we used lentivius particles designed to inhibit expression of miR-122 expression (ABM, Canada). We first explored the optimal time of transduction and MOI. Hepatocytes, Kupffer cells, stellate cells, and endothelial cells were mixed in proportions of 80%, 10%, 5%, and 5%, respectively, and plated in 96 well plates. Each well of 96-well plates contained 1200 cells in total. Different amounts (MOI = 0.5, MOI = 2 and MOI = 5) of lentivirus (10^7^ pfu/mL) expressing Lenti-mir-Off ctrl vector (control) were added to the cells immediately after seeding or after 4 days when the liver organoids are fully formed and compacted. As shown in S3 Fig, the maximum effect was found when the control [ctrl] lentivirus was added immediately after mixing and seeding the cells. In this case, eGFP expression was seen at an MOI of 0.5. GFP. In contrast, when fully formed organoids were transduced eGFP expression could only be detected at MOI’s greater than 5. Therefore, we decided to continue the rest of the experiments with lentiviral infection immediately after cells are mixed.

### miR-122 inhibition in liver organoids leads to liver cell death

**Fig 4:**
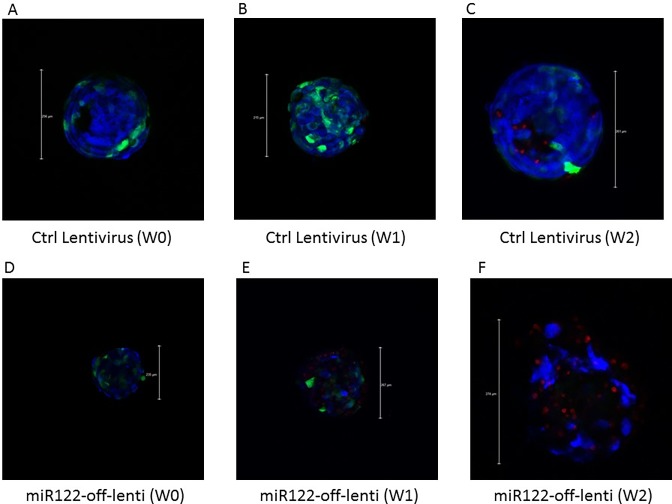
Liver organoid live/dead cell assay. Liver organoids infected with either control lentivirus with MOI = 5 (A-C) or with lentivirus expressing miR-122 inhibitor at different weeks following infection. Organoids were fixed, with calcein Blue and Ethidium homodimer and images were taken using a Leica confocal microscope. Blue = cell nuclei, Red = dead cells, Green = lentivirus GFP expression. W denotes week.

As shown in S4 Fig, using ATP assays as a measure of cell count and viability we did not see a significant change in the number of dead cells at day 4 when we compared organoids transduced with inhibitor virus (Lenti-mir122-Off), control lentivirus and non-transduced organoids. After 7 and 14 days 55% and 63% of cells respectively died after following Lenti-mir122 inhibition (MOI = 2) vs 0% and 20% of cells in control transduced organoids [S4 Fig, (p<0.01)]. Also, at MOI = 3, 60% and 70% of cells died after 1 and 2 weeks following mir122 inhibition vs 0% and 12% of cells in control organoids [S4 Fig, (p<0.01). Finally, at MOI = 5, 65% and 82% of cells died after 1 and 2 weeks following Lenti-mir122-Off infection vs 16% and 21% of cells in control organoids [S4 Fig, (p<0.01)]. Results of live vs dead cell assays detected with confocal microscopy (Fig 4) are consistent with this result. Hence, our preliminary results showed that miR-122 inhibition (at MOI = 5) in liver organoids leads to the death of a large number of liver cells within the organoids.

**Fig 5:**
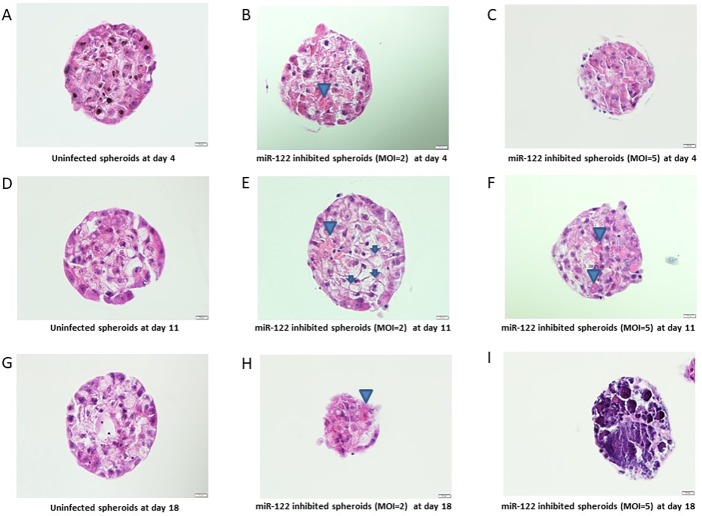
H&E staining of liver organoids. IHC sections of liver organoids stained with H&E at 4 days post-infection (A-C), 11 days post-infection (D-F), and 18 days post-infection (G-I). Left column pictures (A, D, G) show control organoids, middle column show miR-122 inhibited organoids with MOI = 2 (B, E, H), right column show miR-122 inhibited organoids with MOI = 5 (C, F, I). Arrows indicate ballooned hepatocytes, arrowhead indicate Mallory’s hyaline.

Immunohistochemistry was used to detect whether miR-122 inhibition leads to detectable pathological changes within the liver organoids. Hemotoxylin & Eosin (H&E) stained sections of liver organoids exhibited steatotic changes such as ballooning hepatocytes and Mallory’s hyaline started to show up after one-week post transduction in miR-122 deficient organoids at MOI’s of 2 and 5. Such changes were not seen in control transduced organoids (Fig 5E and 5F). In addition, H&E sections of miR-122 deficient organoids at MOI = 5 showed a massive liver organoid cell death which was evident by cell debris on sections (Fig 5I).

### miR-122 inhibition leads to liver fibrosis and steatosis within liver organoids

**Fig 6:**
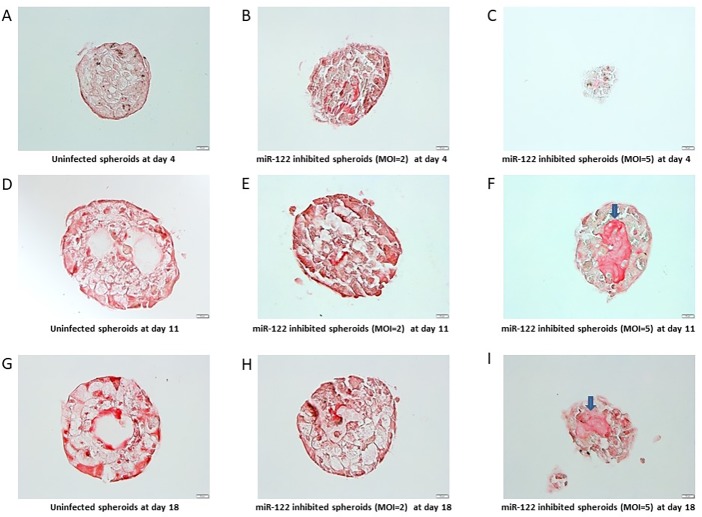
Sirius red staining of liver organoids. IHC sections of liver organoids stained with Sirius Red at 4 days post-infection (A-C), 11 days post-infection (D-F), and 18 days post-infection (G-I). Left column pictures (A, D, G) show control organoids, middle column show miR-122 inhibited organoids with MOI = 2 (B, E, H), right column show miR-122 inhibited organoids with MOI = 5 (C, F, I). Arrows indicate fibrosis.

We also investigated for any histological signs of fibrosis and found that miR-122-inhibited organoids had significantly increased amounts of fibrosis, compared with uninfected organoids at days 11 and 18 post-infection. As shown in Fig 6, fibrosis was more prominent when higher amounts of miR-122 inhibitor was used (MOI = 5) at both days 11 and 18 post-infection compared with uninfected control organoids (Fig 6F and 6I).

### miR-122 inhibition leads to significant alteration in the expression pattern of key genes in inflammation, fibrosis, lipid metabolism and insulin signaling

**Fig 7:**
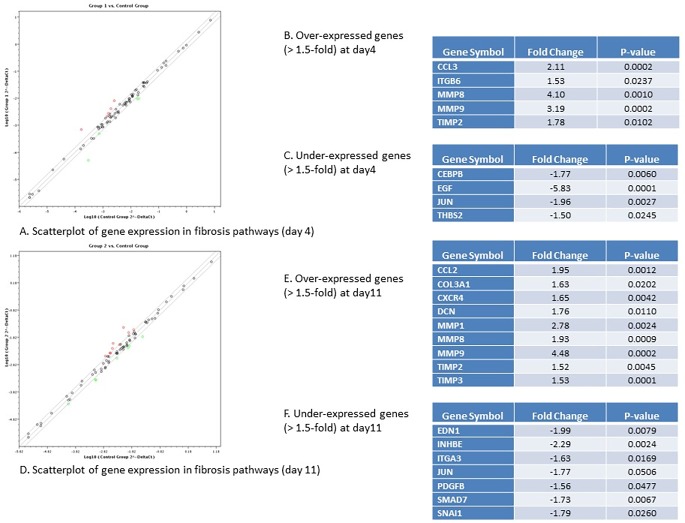
Key genes in fibrosis pathways differentially expressed in liver organoids with miR-122 inhibition vs control organoids. A. Scatterplot of gene expression in fibrosis pathway in miR-122 inhibited organoids after 4 days (group 1) vs control organoids infected with control lentivirus after 4 days (control group). Over-expressed genes are depicted in red while under-expressed genes depicted in green. B. Over-expressed genes (>1.5 fold) at day 4 post-infection. C. Under-expressed genes (<1.5 fold) at day 4 post-infection. D. Scatterplot of gene expression in fibrosis pathway in miR-122 inhibited organoids after 11 days (group 2) vs control organoids infected with control lentivirus after 11 days (control group). Over-expressed genes are depicted in red while under-expressed genes depicted in green. B. Over-expressed genes (>1.5 fold) at day 11 post-infection. C. Under-expressed genes (<1.5 fold) at day 11 post-infection.

Based on our observation of evidence of fibrosis and steatosis in miR-122 deficient liver organoids, we went further and did gene expression profiling on both fibrosis and steatosis panels respectively on the liver organoids with miR-122 inhibition compared with those infected with control lentivirus. The first RT-PCR array, which was done on key genes that are important in the process of fibrosis, showed that there are at least 5 key genes in fibrosis pathways that are over-expressed (more than 1.5-fold) and 4 key genes that are under-expressed (more than 1.5-fold) after 4 days of miR-122 inhibition (Fig 7A–7C), while we found 9 key genes in fibrosis pathways that are over-expressed (more than 1.5-fold) and 7 key genes that are under-expressed (more than 1.5-fold) after 11 days of miR-122 inhibition (Fig 7D–7F). Among the genes that were significantly over-expressed were two inflammatory chemokines CCL3 and CCL2, which were two-fold over expressed after 4 days (Fig 7B) and 11 days (Fig 7E) of miR-122 inhibition. Also, MMP8 and MMP9 and their inhibitor TIMP2 were highly over-expressed (4-fold, 3-fold and 1.7-fold respectively) following 4 days of miR-122 inhibition, and they constantly remained over-expressed after 11 days (Fig 7B and 7E). We found EGF as the most significantly down-regulated gene after 4 days of infection which its expression was 5.8 times reduced. Among the genes under-expressed after 11 days post-infection, INHBE was found to be the most significantly down-regulated genes with 2.3-fold decrease in expression. We also found slight increase in COL3A1 and CXCR4 both of which play important roles in hepatic fibrosis.

**Fig 8:**
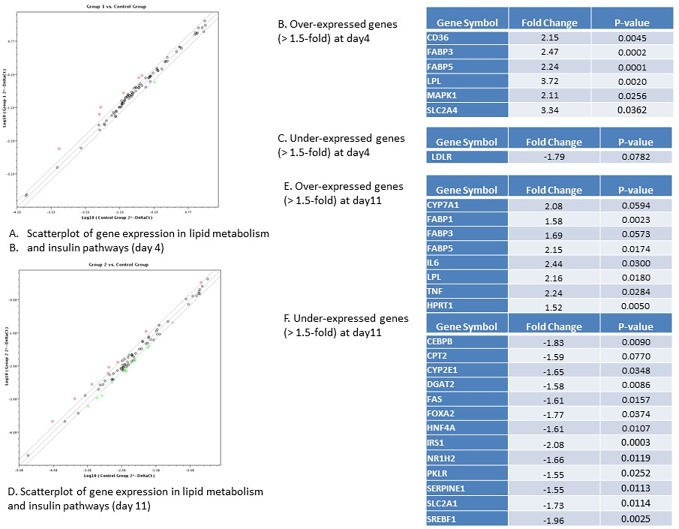
Key genes in lipid metabolism and insulin signaling pathways differentially expressed in liver organoids with miR-122 inhibition vs control organoids. A. Scatterplot of gene expression in lipid metabolism and insulin signaling pathway in miR-122 inhibited organoids after 4 days (group 1) vs control organoids infected with control lentivirus after 4 days (control group). Over-expressed genes are depicted in red while under-expressed genes depicted in green. B. Over-expressed genes (>1.5 fold) at day 4 post-infection. C. Under-expressed genes (<1.5 fold) at day 4 post-infection. D. Scatterplot of gene expression in lipid metabolism and insulin signaling pathway in miR-122 inhibited organoids after 11 days (group 2) vs control organoids infected with control lentivirus after 11 days (control group). Over-expressed genes are depicted in red while under-expressed genes depicted in green. B. Over-expressed genes (>1.5 fold) at day 11 post-infection. C. Under-expressed genes (<1.5 fold) at day 11 post-infection.

We also performed qPCR arrays on key genes in lipid metabolism and insulin signaling. We found that six genes in these pathways are over-expressed (more than 1.5 fold) and only one gene was under-expressed (more than 1.5 fold) after 4 days of miR-122 inhibition (Fig 8A–8C). The numbers were increased to 8 and 15 genes which were over- and under-expressed after 11 days of miR-122 inhibition (Fig 8D). Lipoprotrein Lipase (LPL) and SLC2A4 (Glut4) were among the most significantly over-expressed genes after 4 days of miR-122 inhibition with 3.7- and 3.3-fold increase in gene expression respectively while LDLR was found the only under-expressed gene with 1.7-fold decrease in its expression (Fig 8B and 8C). Among over-expressed genes IL-6 and TNFα –both inflammatory cytokines) were found to have the most significant over-expression with 2.4 and 2.2-fold over-expression (Fig 8E). We found 15 genes were down-regulated (more than 1.5-fold) after 11 days of miR-122 inhibition which represented a major portion (18%) of all genes which were examined by qPCR (Fig 8D–8F). Among these, we found IRS1, SREBF1, and FoxA2 with 2-fold down-regulation are the most significantly under-expressed genes. All 15 under-expressed genes playing important roles in insulin signaling and/or lipid metabolism as well as adipokine signaling (Fig 8F).

## Discussion

We constructed liver organoids *in vitro* containing four different main primary human liver cells: hepatocytes, Kupffer cells, hepatic stellate cells and sinusoidal endothelial cells. The organoids were fully formed after 4 days and stayed more than 90% viable over four weeks in culture. By immunohistochemistry, we showed that they stored glycogen, make tight junctions and Gap junctions and that non-parenchymal cells are present tightly interspersed with hepatocytes. We were also able to show the presence of rudimentary bile canaliculi between adjacent hepatocytes, indicating the establishment of a primitive biliary system. In addition, they maintained measurable drug metabolism (CYP3A4 and CYP2C19) over 28 days in contrast to 2D cultures in which drug metabolism was only detectable up to 7 days despite containing almost 100 times more cells [15,16].

After constructing our liver organoid model, we showed that miR-122 inhibition leads to inflammation, necrosis, steatosis and fibrosis. We found out that 65% and 82% of cells died after 1 and 2 weeks following mir122 inhibition by transduction with Lenti-mir122-Off (MOI = 5) vs 16% and 21% of cells in control organoids. Also, miR-122 deficient liver organoids showed a higher expression of inflammatory markers like CCL2, CCL3, TNF and IL-6 as well as ECM molecules like MMP1, MMP8, MMP9, TIMP2, TIMP3 and altered expression of key genes in lipid metabolism and insulin signaling. We also found histological evidence of fibrosis and steatosis when miR-122 was highly down-regulated.

Our results are in accordance with animal model which showed that Mir122a-knock-out animals develop steatohepatitis and liver fibrosis [17,18]. These studies also demonstrated an increase in infiltrating inflammatory cells in miR-122–deficient mice liver similar to what we observed in miR-122-deficient liver organoids [18]. They also showed that inflammatory cells in miR-122–deficient mice liver produce high levels of IL-6 and TNF-α, and these cells are known to promote fibrosis in the injured liver-the exact molecular pathology mechanisms we observed in our miR-122 deficient liver organoids. MiR-122 was reported to be significantly decreased in steatohepatitis in the fibrotic liver of mice fed with a methionine-choline-deficient diet for eight weeks [18]. Recently, another study demonstrated miR-122 serum levels were robustly increased early on in a diet-induced model of NAFLD and correlated with ALT and AST levels, and supports the notion that reductions in hepatocytic miR-122 correlates to and can indicate inflammation in the setting of NASH [19]. Result of our study further support this notion that miR-122 deficiency in patients with NASH is a key mechanism for induction of liver inflammation, necrosis, and steatofibrosis.

Different types of immune cells are recruited and/or activated to the site of injury, contributing to NASH development and progression. Kupffer cell (KC) activation is critical in NASH and precedes the recruitment of other cells [20]. When KCs develop a pro-inflammatory M1 phenotype, it leads to the secretion of various pro-inflammatory and pro-fibrogenic cytokines, such as IL-1b, IL-12, TNF-α, CCL2, CCL3 and CCL5. Induction of these cytokines and chemokines can result in further hepatocyte damage and release of damage-associated molecular patterns (DAMPs). DAMP release can induce further KC activation and inflammation as well as HSC activation and initiating a fibrogenic response [21]. Our results highlight the importance of inflammation in the process of liver necrosis due to down-regulation of miR-122. Both CCL2 and CCL3 have been recently identified among the key cytokines altered in NASH. CCL2 was found to be elevated in patients with NASH [22]. It is also known that CCL3 is correlated with serum aminotransferase levels and the histological severity of NASH [23]. Following tissue injury, kupffer cells and other hepatic cells (hepatocytes and HSCs) secrete chemokines like CCL2 and CCL3 which induces massive infiltration of monocytes to injured liver as well as initiation of a fibrogenic response through HSC activation. To identify which hepatic cell plays a critical role in DAMP release and cell death following miR-122 inhibition, we decided to compare organoid viability among organoids with different mixing of cells through excluding KCs, or HSCs or both cells from organoid co-culture. Interestingly, we observed that miR-122 abrogation leads to liver cell death only when both hepatocytes and KCs exist in organoid co-culture (Data not shown) irrespective of HSCs. This means that hepatocytes and KCs are the key cells for initiation of cell death process following miR-122 inhibition. We believe that this is mainly because hepatocytes are considered to be main hepatic cell expressing miR-122 within liver tissue, and kupffer cells play more important role in promoting the inflammation following hepatocyte death. However, we need to emphasize that the role of HSCs in inducing fibrogenic process following DAMP release, should not be overlooked.

We also found increased levels of IL6 and TNF in miR-122 deficient liver organoids similar to what was found in miR-122 deficient mice. Hepatic IL-6 expression is known to be elevated in the livers of patients with NASH as compared to patients with simple steatosis or normal liver biopsies [24]. Our data, in accordance with previous studies, suggests that increased hepatic IL-6 production plays an important role in steatofibrosis, as well as in systemic insulin resistance since adipose tissue-derived IL-6 has been shown to regulate hepatic insulin resistance [25]. Also, the importance of TNF-α in human and animal fatty liver diseases, both due to genetic manipulation and over-nutrition, has been demonstrated. Furthermore, neutralization of TNF-α activity improves insulin resistance and fatty liver disease in animals [26]. In accordance with previous studies, we also found increased levels of TNF in our one week liver organoids. The lack of immediate increase in both IL6 and TNF following miR-122 inhibitor infection shows that these cytokines possibly are not among direct targets of miR-122 and miR-122 inhibition increases these cytokines through indirect effect on some other elements of hepatic signal transduction.

At the histological level, miR-122-inhibited organoids had significantly increased amounts of fibrosis at days 11 and 18 post-infection compared with uninfected organoids. Histological findings of liver fibrosis in our miR-122 deficient organoids were associated with increased expression of collagen III. Interestingly we also found that MMP8 and MMP9 and their inhibitors TIMP2 and TIMP3 were highly overexpressed (4-fold, 3-fold and 1.7-fold, 1.5-fold respectively) upon miR-122 inhibition in liver organoids. Regression of liver fibrosis is known to be associated with increased matrix degradation. For liver fibrosis to resolve, the expression of TIMPs rapidly decreases while interstitial MMPs continue to be expressed during the resolution, resulting in increased MMP activity and consequent matrix degradation within the liver [27]. In our miR-122 inhibited organoids, TIMP2 and TIMP3 expression still remains high at day 11 post-infection while MMP8 decreases more than 2- fold at day 11 vs day 4 post-infection though we see a slight increase in the levels of MMP1 and MMP9 at day 11 compared with day 4. Perhaps the imbalance between increase in the expression of some of the MMPs and increase in their inhibitor (TIMPs) is the main reason that liver fibrosis persists. For the resolution of miR-122 deficiency-induced liver fibrosis, we anticipate that an earlier, stronger and constant increase in selected MMPs and a decrease in TIMPs could lead to the resolution of liver fibrosis in liver organoids and this may work as well in patients with NAFLD. Besides, the role of HSCs in induction of fibrosis needs to be explored further. In a recent study, Li et al., found that miR-122 regulates collagen production by targeting HSCs and suppressing P4HA1 [28]. Therefore they suggested that miR-122 negatively regulating collagen and ECM production in HSCs. The same phenomena may apply to our liver organoid and HSCs depleted from miR-122 can be transactivated through increase in P4HA1 expression.

miR-122 inhibition is known to decrease serum cholesterol through downregulation of several genes which play role in cholesterol biosynthesis [29] like what we observed such as a mild (30%) reduction in expression of hepatic HMG CoA reductase (HMGCR) at 4 days post-infection. Interestingly, we also observed a two-fold increase in expression of CYP7A1 at 4 days post-infection. CYP7A1 is the rate-limiting enzyme in the synthesis of bile acid from cholesterol. CYP7A1 is up-regulated by the nuclear receptor liver LXR when cholesterol levels are high [30]. The effect of this up-regulation is to increase the production of bile acids and reduce the level of cholesterol in hepatocytes. Our results, which show a decrease in HMGCR and an increase in CYP7A1 expression following miR-122 inhibition indicates decrease in hepatic cholesterol levels could be secondary to miR-122 inhibition. These results are in accordance with previous animal studies which showed miR-122 is a key regulator of cholesterol metabolism. In addition, we also found a 2.2- fold increase in CD36 which is known to work as an HDL receptor, as well as a 1.8-fold decrease in expression of LDL receptor. Although the serum lipid profiles of both liver-specific knockouts (LKO) and germline knockouts (KO) show a 30% reduction in total cholesterol, LDL, HDL, and serum triglyceride (TG), the livers of both KO and LKO mice also have progressive steatohepatitis [17,18].

Lipoprotein Lipase (LPL) is a key enzyme with important roles in both metabolism and transport of lipids. LPL is responsible for the hydrolysis of Triglyceride (TG) which exists in circulating chylomicrons and VLDLs [31]. The importance of LPL in adult liver is not well known because its expression level is quite low. Nevertheless, it is known that overexpression of LPL in hepatocytes can result into a 2-fold increase in liver TG content and insulin resistance in mice. This effect is known to be due to inability of insulin to suppress endogenous glucose production and it is associated with impairments of insulin activation of insulin receptor substrate-2-associated phosphatidylinositol 3-kinase activity [32]. Our results are in accordance with these studies and shows that miR-122 inhibition in the liver leads to lipid accumulation and dysregulation of insulin signaling possibly through a 3.7-fold increase in LPL expression. Interesting, we also found a 3.3-fold increase in expression of Glucose transporter-4 (GLUT4). GLUT4, which is considered to be one of the most important glucose transport isoforms in regard to responding to insulin, plays a critical role in transporting glucose from outside of cells into insulin-sensitive cells and its expression is mostly in skeletal muscle and adipose tissues. Although there are few reports on the hepatic expression of GLUT4 in liver cells, but it has been reported that stellate cells and endothelial cells can express GLUT4 [33]. Whether our GLUT4 signal comes from hepatocytes, stellate cells or endothelial cells is not clear but other have shown that over-expression of GLUT4 prevented the normal induction of *SREBP-1c* and *FAS* expression, suggesting that normal lipogenesis is altered in these transgenic animals [34]. Interestingly we found a similar suppression of SREBP-1 and FAS one week after over-expression of GLUT4 and LPL. Apart from SREBP-1C and FAS, we found a list of 13 other key genes important in insulin signaling or lipid metabolism suppressed one week after initial increase in LPL and GLUT4.

Insulin resistance can be monitored by the decreased phosphorylation of two main substrates of the insulin receptor, IRS1 and IRS2. Both of these proteins signal through PI3K, Akt and their downstream targets [35]. Our results show a 2.1- fold decrease in IRS1 expression. Interestingly, we also found a 1.8-fold decrease in the expression levels of FoxA2, which is a transcription factor downstream of IRS and PI3K/AKT pathway and regulates lipid metabolism and ketogenesis in the liver during fasting [36]. It is known that chronic hyperinsulinemia in insulin-resistant syndromes results in the cytoplasmic localization and inactivation of FoxA2, thereby promoting lipid accumulation and insulin resistance in the liver [36]. We believe that 1.8-fold decrease in the expression of FoxA2 after miR-122 inhibition can further increase lipid accumulation and insulin resistant in the liver.

We also observed an increase in the expression of FABP-1, FABP-3 and FABP-5. Similarly, Liver FABP levels in NAFLD patients were known to be higher than in the control group, and a strong correlation was found between serum L-FABP concentrations and liver transferases, body mass index, glucose and γ-glutamyltransferase levels [37]. Interestingly, mice expressing high levels of FABP-5 in adipose tissue display reduced systemic insulin sensitivity as well.

To summarize the effects of miR-122 inhibition on lipid metabolism and insulin signaling, we showed that miR-122 inhibition leads to an increase in LPL, GLUT4 and FABPs at 4 days post-infection of miR-122 inhibitor. The increase in hepatic LDL can lead to an increase in lipid (TG) accumulation which was evident by ballooning hepatocytes and Mallory’s Hyaline deposition as early as 4 days post-infection. MiR-122 inhibition also leads to an increase in expression of GLUT4 and a consequent increase in transport of glucose into the hepatocytes and this leads to desensitizing insulin receptor as it is evident by decrease in expression of IRS1 and several other molecules (SREBP1, FAS, FoxA2) under insulin signaling in one hand and increase in release of inflammatory cytokines (IL6, TNFα) and chemokines (CCL2, CCL3, CXCR4) from Kupffer cells due to hepatocyte death as was seen after 11 days post-infection.

In summary, we found that miR-122 inhibition in liver organoids leads to liver inflammation, necrosis, fibrosis, steatosis as well as dysregulation in lipid metabolism as well as insulin signaling. As patients with NAFLD are known to have altered levels of miR-122; and as most of NAFLD patients have some degree of insulin resistance, our results highlight the role of miR-122 in insulin sensitivity suggests that miR-122 mimics could play a useful role in reversing liver inflammation, steatofibrosis and insulin resistant seen in these patients.

## Supporting information

S1 Fig Drug metabolism in 2D and 3D culture systems: Mass spectrometery quantification of the diazepam metabolites A) temazepam, B) noridazepam, and C) oxazepam primarily by CYP2C19 and CYP3A4. 2 Liver organoids were found to have measurable cytochrome P450 drug metabolism activity for at least 28 days, in comparison to standard monolayer sandwich cultures that lost CYP450 activity after 7 days Statistical significance: * p < 0.05 between 3-D and 2-D comparison at each time point.
(TIF)

S2 Fig Toxicity assays in liver oraganoids. (A) 14D liver organoids were treated with increasing doses of troglitazone for 48 hours and viability assessed by cellular ATP content. The EC50 value was approximately 100uM. (B) Shows the intracellular accumulation of neutral lipids after troglitazone insult as a model for liver steatosis. (D)intracellular accumulation of phospholipids modelling liver phospholipidosis.
(TIFF)

S3 Fig GFP expression as an index to compare the infection of liver organoids with control lentivirus. (A-C) shows liver organoid when lentivirus immediately added after mixing of cells. D. Shows a liver organoid when lentivirus is added after organoid is made (at 4 days after co-culturing). Green color represents cells infected with lentivirus. MOI stands for Multiplicity of Infection.
(TIFF)

S4 Fig ATP assay in liver organoids. The results of ATP assay are shown as the percentage of live cells in miR-122 inhibited organoids (miR-122-off) or organoids infected with lentivirus (ctrl-v) compared with uninfected organoids (considered 100%). Organoids were infected with different MOIs (2,3, 5) at 4days (A), 11 days(B) and 18 days (C) post-infection.
(TIFF)
