# Supplementary figures and images for: miR-122 inhibition in a human liver organoid model leads to liver inflammation, necrosis, steatofibrosis and dysregulated insulin signaling
Source: PLoS One. 2018 Jul 19;13(7):e0200847. doi: 10.1371/journal.pone.0200847 (PMC6053181; doi:10.1371/journal.pone.0200847)

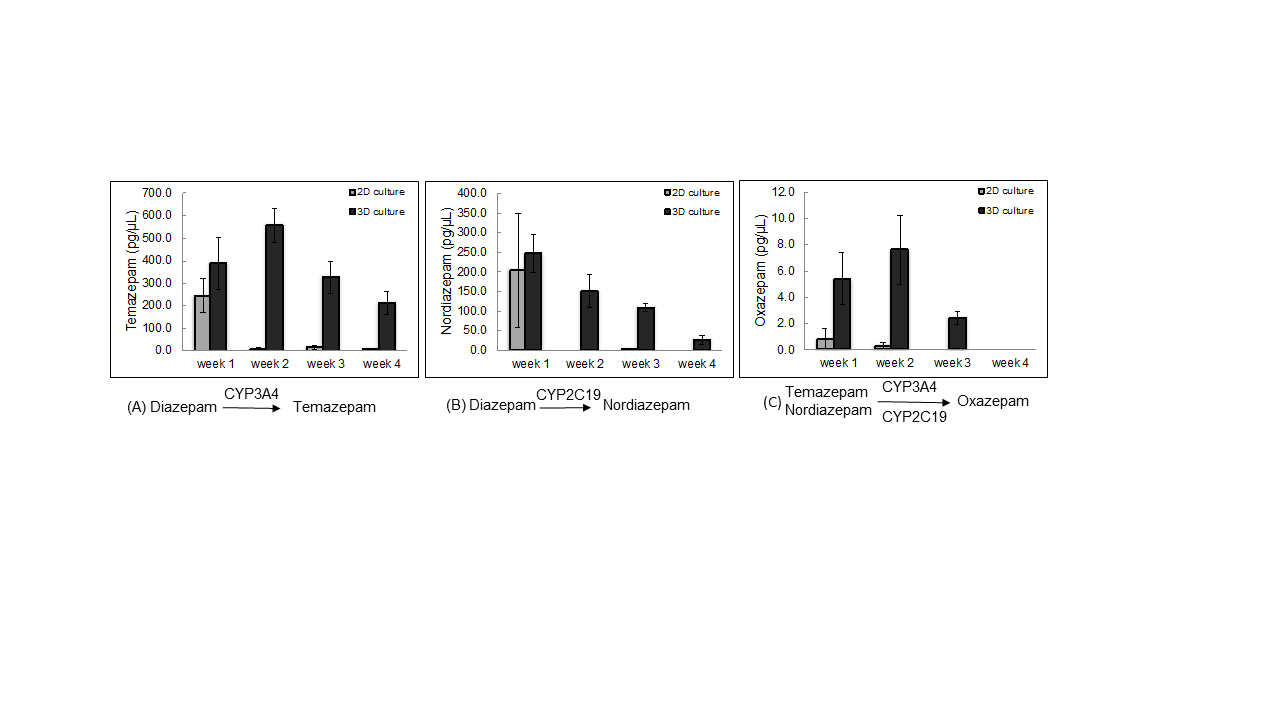

Supplement: S1 Fig — Drug metabolism in 2D and 3D culture systems: Mass spectrometery quantification of the diazepam metabolites A) temazepam, B) noridazepam, and C) oxazepam primarily by CYP2C19 and CYP3A4. 2 Liver organoids were found to have measurable cytochrome P450 drug metabolism activity for at least 28 days, in comparison to standard monolayer sandwich cultures that lost CYP450 activity after 7 days Statistical significance: * p < 0.05 between 3-D and 2-D comparison at each time point. (TIF) [file pone.0200847.s001.tif]

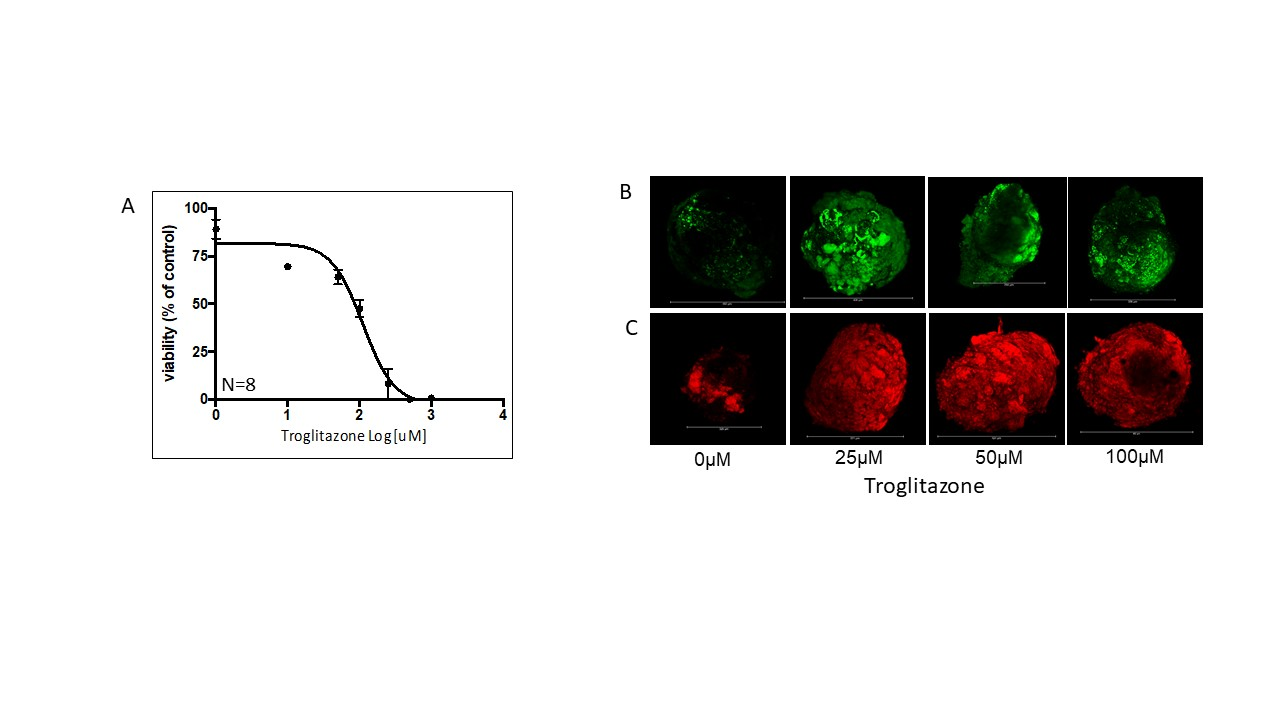

Supplement: S2 Fig — (A) 14D liver organoids were treated with increasing doses of troglitazone for 48 hours and viability assessed by cellular ATP content. The EC50 value was approximately 100uM. (B) Shows the intracellular accumulation of neutral lipids after troglitazone insult as a model for liver steatosis. (D)intracellular accumulation of phospholipids modelling liver phospholipidosis. (TIFF) [file pone.0200847.s002.tiff]

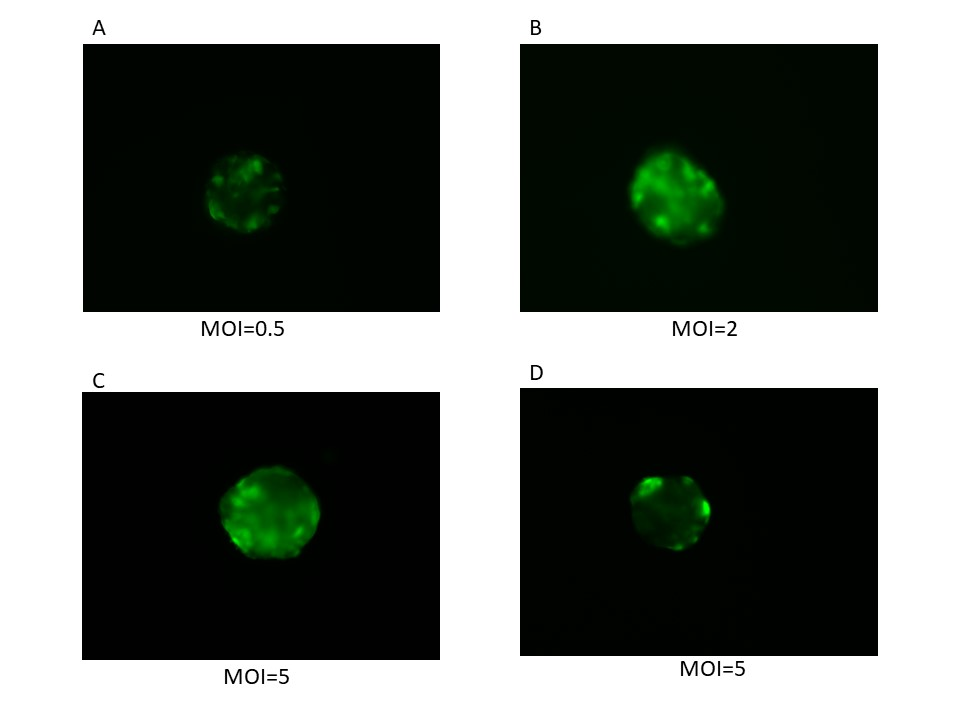

Supplement: S3 Fig — (A-C) shows liver organoid when lentivirus immediately added after mixing of cells. D. Shows a liver organoid when lentivirus is added after organoid is made (at 4 days after co-culturing). Green color represents cells infected with lentivirus. MOI stands for Multiplicity of Infection. (TIFF) [file pone.0200847.s003.tiff]
